# Molecular cytogenetic and morphological characterization of two wheat-barley translocation lines

**DOI:** 10.1371/journal.pone.0198758

**Published:** 2018-06-11

**Authors:** László Ivanizs, András Farkas, Gabriella Linc, Márta Molnár-Láng, István Molnár

**Affiliations:** 1 Agricultural Institute, Centre for Agricultural Research, Hungarian Academy of Sciences, Martonvásár, Brunszvik u. 2, Hungary; 2 Institute of Experimental Botany of the Czech Academy of Sciences (IEB), Centre of the Region Haná for Biotechnological and Agricultural Research, Šlechtitelů 31, Olomouc-Holice, Czech Republic; Institute of Genetics and Developmental Biology Chinese Academy of Sciences, CHINA

## Abstract

Barley chromosome 5H, carrying important QTLs for plant adaptation and tolerance to abiotic stresses, is extremely instable in the wheat genetic background and is eliminated in the early generations of wheat-barley crosses. A spontaneous wheat-barley 5HS-7DS.7DL translocation was previously obtained among the progenies of the Mv9kr1 x Igri hybrid. The present work reports on the transfer of the 5HS-7DS.7DL translocation into a modern wheat cultivar, Mv Bodri, in order to use it in the wheat breeding program. The comparison of the hybridization bands of DNA repeats HvT01, pTa71, (GAA)_n_ and the barley centromere-specific (AGGGAG)_n_ in Igri barley and the 5HS-7DS.7DL translocation, together with the visualization of the barley chromatin made it possible to determine the size of the introgressed barley segment, which was approximately 74% of the whole 5HS. Of the 29 newly developed PCR markers, whose source ESTs were selected from the Genome Zipper of barley chromosome 5H, 23 were mapped in the introgressed 1–0.26 FL 5HS bin, three were located in the missing C-0.26 FL region, while three markers were specific for 5HL. The translocation breakpoint was flanked by markers Hv7502 and Hv3949. A comparison of the parental wheat cultivars and the wheat-barley introgression lines indicated that the presence of the translocation improved tillering ability in the Mv9kr1 and Mv Bodri genetic background. The similar or better yield components under high- or low-input cultivation environments, respectively, indicated that the 5HS-7DS.7DL translocation had little or no negative effect on yield components, making it a promising genotype to improve wheat genetic diversity. These results promise to accelerate functional genomic studies on barley chromosome 5H and to support pre-breeding and breeding research on wheat.

## Introduction

The genetic diversity of common wheat (*Triticum aestivum* L.) can be extended via interspecific hybridization. One of the promising crossing partners is barley (*Hordeum vulgare* L.), which has many agronomically useful traits, such as earliness [1], tolerance of biotic [2,3] and abiotic (Al and salt) stress [4–7], good tillering ability [8,9] and high grain protein [10] and dietary fiber content [11,12], which it would be desirable to transfer into wheat.

The first wheat-barley hybrids and addition lines (2H, 3H, 4H, 5H, 6H, 7H) were obtained from crosses between wheat genotype Chinese Spring and spring barley genotype Betzes [13].

In order to produce introgressions with higher breeding value, the winter wheat line Mv9kr1, which has good crossability characters, and the German two-rowed winter barley genotype Igri were crossed to produce new wheat-barley hybrids and disomic addition lines (2H, 3H, 4H, 6HS, 7H, 1HS isochromosome) [14]. Unfortunately, the barley chromosome 5H was not represented in either set of addition lines, because the 5H chromosome is eliminated at high frequency in the early generations of wheat-barley crosses [15,16]. On the other hand, it would be highly desirable to utilize the genetic potential of barley chromosome 5H in wheat breeding, because several important QTLs responsible for salt and drought tolerance have been mapped on the short arm of this chromosome [17,18].

Other studies also reported the extremely high elimination frequency of barley chromosome 5H [13,19,20], which makes it very difficult to develop a wheat-barley introgression line involving the 5H chromosome. The spontaneous, non-compensating wheat-barley translocation selected by genomic *in situ* hybridization (GISH) from Mv9kr1 x Igri progenies [21] is thus of great importance. The 5HS-7DS.7DL translocation was identified by Nagy et al. [22] using fluorescence *in situ* hybridization (FISH) and 5H barley chromosome-specific SSR (Simple Sequence Repeat) markers. It was found that the proximal half of the 5HS arm is missing from the translocation [22]. The wheat 7DS chromosome arm involved in this translocation was also characterized using physically mapped SSR markers in order to determine the size of the deleted 7DS fragment [23]. The absence of certain 7DS-specific marker products indicated the elimination of the terminal region of the 7D chromosome [23].

Although Mv9kr1 is an easily crossable wheat genotype, containing the major crossability gene *Kr1* transferred from Chinese Spring in recessive homozygous form (*kr1kr1*) [24], it has some disadvantageous traits, such as larger plant height and sensitivity to diseases due to the presence of Chinese Spring alleles. The second step in the utilization of wheat-alien introgression lines in breeding programs is the transfer of the translocated chromosome into an elite wheat genotype.

The linear gene order (Genome Zipper) of the barley chromosome 5H contains mapped ESTs [25,26] which may have insertion/deletion (InDel) polymorphisms relative to wheat. These InDel polymorphisms can be used as gene-specific molecular markers for the precise mapping of barley chromosome segments in the wheat background as well as to support introgression breeding by the marker-assisted selection of wheat-barley introgression lines.

In the present work, newly developed EST-derived InDel markers were used, together with *in situ* hybridization techniques, to precisely characterize the structure of the barley 5HS chromosome segment transferred into Mv Bodri. The effect of the 5HS-7DS.7DL translocation on the agronomic traits of Mv Bodri was compared with its effect in the original crossing partner Mv9kr1.

## Material and methods

### Plant material

A spontaneous 5HS-7DS.7DL translocation was produced from a hybrid between the winter wheat line Mv9kr1, and the barley cultivar Igri [21]. The translocation line, described as 5HS-7DS.7DL/Mv9kr1, was maintained by self-fertilization and crossed with a modern winter wheat cultivar, Mv Bodri, which has good agronomic performance. The F_2_ genotype carrying the translocation in disomic form was selfed three times to obtain a sufficient number of uniform progenies for agronomic investigation. These genotypes, containing a mixed wheat genetic backround originating from Mv9kr1 and Mv Bodri, were designated as 5HS-7DS.7DL/Mv9kr1/Mv Bodri.

The barley cultivar Betzes (*Hordeum vulgare* L., 2n = 2x = 14) and the Chinese Spring-Betzes wheat–barley 5H disomic addition line [13] were used as positive controls and the wheat genotype Chinese Spring as negative control for the PCR validation of EST-derived markers.

### Field trials and agronomic investigation of plants

Agronomic investigation of the genotypes was carried out in a low-input field (Tükrös Nursery, Martonvásár, Hungary; geographic coordinates: 47°18'40"N 18°46'56"E) in the 2015–2016 season and in a high-input location (Breeders nursery, Martonvásár, Hungary; 47°19'58"N 18°47'08"E) in 2016–2017. As both areas are owned by the Agricultural Institute, Centre for Agricultural Research, Hungarian Academy of Sciences, no specific permission was required for the experiments. It is also confirmed that the field studies did not involve endangered or protected species.

The genotypes 5HS-7DS.7DL/Mv9kr1, 5HS-7DS.7DL/Mv9kr1/Mv Bodri, Mv9kr1 and Mv Bodri were grown in chernozem soil in both locations. In the low-input field, which was treated with herbicides [27], each genotype was sown in 5 × 1 m rows with 10 seeds per row and a row distance of 15 cm. In the high input field, each genotype was raised in a 4 m^2^ plot with 6 x 3 m rows, 50 seeds per row, and a row distance of 20 cm. Ten typical plants per genotype were randomly selected and analyzed for agronomic traits. Plant height and tillering were recorded directly before harvest, while seeds/spikelet, length of the main spike, number of seeds/main spike, number of spikelets/main spike and number of seeds/plant were assessed after harvest.

### *In situ* hybridization

Root tip chromosome preparations of the genotypes 5HS-7DS.7DL/Mv9k1, 5HS-7DS.7DL/Mv9kr1/Mv Bodri and Igri were made according to Jiang et al. [28].

Total genomic DNA was isolated from the barley genotype Igri using a DNA isolation kit (FujiFilm, Japan) and labeled with digoxigenin-11-dUTP (Roche Diagnostics, Mannheim, Germany) by nick translation. The HvT01 repeat was amplified from barley genomic DNA using PCR and labeled with digoxigenin-11-dUTP (Roche, Mannheim, Germany) [29]. The pTa71 probe was isolated from wheat [30] labeled with 50% biotin-16-dUTP and 50% digoxigenin-11-dUTP by nick translation (Roche). The (GAA)_n_ microsatellite was amplified from barley [31] and labeled with biotin-16-dUTP (Roche) by PCR according to Vrána et al. [32], and the barley centromere-specific probe (AGGGAG)_n_ was labeled with digoxigenin-11-dUTP by PCR as described by Hudakova et al. [33].

GISH was carried out on genotypes 5HS-7DS.7DL/Mv9k1 and 5HS-7DS.7DL/Mv9kr1/Mv Bodri as described by Reader et al. [34] with minor modifications [35]. For the detection of the hybridization signals, 10 μg mL^-1^ each of streptavidin-FITC (Roche) and anti-digoxigenin-rhodamin (Roche) were used. The chromosomes were counterstained with 2 μg mL^-1^ DAPI (4′,6-diamidino-2-phenylindole, Amersham, Bucks., UK) and the slides were mounted in Vectashield fading inhibitor solution (Vector Laboratories, Burlingame, USA).

After documentation of the GISH sites, the slides were washed and rehybridized using the repetitive DNA probes HvT01, pTa71 and (GAA)_n_ and the barley centromere-specific sequence (AGGGAG)_n_. The FISH experiments was carried out according to the methods of Linc et al. [36] with modifications as described by Szakács and Molnár-Láng [14]. Pretreatments and stringency washings before the FISH experiments on the barley chromosome preparations were the same as those used for GISH and the same hybridization solutions containing repetitive DNA probes HvT01, pTa71, (GAA)_n_ and (AGGGAG)_n_ were used as for the 5HS-7DS.7DL translocation lines.

Mitotic cells were examined with a Zeiss Axio Imager M2 fluorescence microscope equipped with the appropriate filter sets (Carl Zeiss Mikroskopie, Jena, Germany). Images were captured with a Zeiss AxioCam MRm CCD camera (Diagnostic Instruments, Sterling Heights, Mich., USA) and processed with Zeiss Axiovision 4.8.2. software.

HvT01 is a barley-specific repetitive sequence located on the subtelomeric region of 5HS, pTa71 is an rDNA probe specific for the NOR region and able to detect the position of the secondary constriction on the 5HS arm, the (GAA)_n_ oligonucleotide is located on the pericentric region of 5HS, while the (AGGGAG)_n_ repeat is specific for the barley centromere. The length of the chromosome segments and the distances between the FISH signals were measured using Image Pro Plus 5.1 software. The positions of the translocation breakpoint and each of the FISH signals on the barley chromatin were expressed as fraction length (FL) values from the centromere relative to the length of the complete 5HS arm.

### Development of EST-derived markers and PCR analysis

Genomic DNA was extracted from fresh young leaves (plants in the 2-leaf stage) from wheat genotypes Mv9kr1, Mv Bodri and Chinese Spring, barley cultivars Igri and Betzes, the Chinese Spring-Betzes 5H addition and the 5HS-7DS.7DL/Mv9kr1 and 5HS-7DS.7DL/Mv9kr1/Mv Bodri translocation lines using Quick Gene-Mini80 (FujiFilm, Japan) with a QuickGene DNA tissue kit (FujiFilm, Japan) according to the manufacturer’s instructions [37].

The barley ESTs specific for chromosome 5H were selected from the publicly available barley Genome Zipper (http://pgsb.helmholtz-muenchen.de/plant/barley/gz/tablejsp/index.jsp) [26,38] and then aligned to the genomic sequences of the corresponding wheat homeologous chromosomes using BLASTn (https://urgi.versailles.inra.fr/blast/blast.php) to find ESTs showing polymorphism relative to wheat. BLASTn hits that met certain criteria (at least 7–8 bp InDel polymorphism and at least 80% homology between barley and wheat sequences) were considered as significant. The pairwise alignment of the selected ESTs and wheat contigs was carried out with UGENE software (v.1.23.0) to identify InDel regions between wheat and barley and to design barley-specific primers. The markers specific for barley chromosome 5H were validated by PCR on the genotypes Chinese Spring and Betzes and on the Chinese Spring-Betzes 5H addition lines. Two or three primer pairs were designed for each barley EST (Integrated DNA Technologies, Coralville, Iowa, USA) and the primer pair producing the most typical barley signal was chosen for subsequent analysis. The translocation genotypes were analyzed using primer pairs selected after the validation test.

The PCR reactions were performed in a final volume of 15 μL containing 20 ng of template DNA, 1.5 μL of 10× key reaction buffer (MgCl_2_ final concentration of 1.5 mmol/L), 200 μmol/L of each dNTP, 0.2 μmol/L of forward and reverse primers, and 0.375 U of TEMPase Hot Start DNA Polymerase (VWR International, Belgium). The reactions were carried out in an Eppendorf Mastercycler (Eppendorf, Germany) using the following PCR profile: 15 min at 95°C, 35 cycles of 20 s at 95°C, 20 s at 58°C, 30 s at 72°C, and a final extension at 72°C for 5 min, following the manufacturer’s instructions (VWR International). All the molecular markers were constructed to have the same melting point (58 °C). The PCR products were separated using a Fragment Analyzer™ Automated CE System equipped with a 96-Capillary Array Cartridge (Advanced Analytical Technologies, USA). The separated nucleic acid fragments were visualized as digital capillary electrophoretic gel images, provided by the PROsize v2.0 software (Advanced Analytical Technologies, USA), in order to compare the size and concentration data of all the genotypes investigated.

### Statistical analysis

In order to determine which fragment of 5HS was deleted in the wheat-barley translocation lines 5HS-7DS.7DL/Mv9kr1/Mv Bodri and 5HS-7DS.7DL/Mv9kr1, the length of the chromosome segments and the distances between the FISH signals were measured in 20 chromosomes from 5HS-7DS.7DL/Mv9kr1, 40 from 5HS-7DS.7DL/Mv9kr1/Mv Bodri and 30 from barley cultivar ‘Igri’ using Image Pro Plus 5.1 software.

The amount of introgressed barley chromatin was compared in the two introgression lines 5HS-7DS.7DL/Mv9kr1/Mv Bodri and 5HS-7DS.7DL/Mv9kr1 using Student’s t-tests for paired data at the P = 0.05 significance level.

The agronomic traits of lines 5HS-7DS.7DL/Mv9kr1/Mv Bodri and 5HS-7DS.7DL/Mv9kr1 and of parental wheat cultivars Mv9kr1 and Mv Bodri were compared pair-wise with each other. Differences in agronomic traits between the genotypes were evaluated by means of Tukey’s post hoc test (SPSS 16.0) at the P = 0.05 significance level.

## Results

### Transfer of the 5HS-7DS.7DL translocation into a modern wheat cultivar, Mv Bodri

In order to investigate the effect of the 5HS-7DS.7DL translocation on the agronomic performance of an elite wheat cultivar and to introduce this translocation into wheat breeding programs, a cross was made between 5HS-7DS.7DL/Mv9kr1 and Mv Bodri. The inheritance of the translocation was traced by GISH in 40 F_2_ progenies of this cross. Among the F_2_ genotypes the barley chromosome was eliminated from 27 plants, while 12 progeny carried the translocation in monosomic form and one in disomic form. As the 5HS-7DS.7DL/Mv9kr1 genotype was used as a crossing partner, the barley chromosome segment was regarded as originating from the 5HS-7DS.7DL translocation.

### Physical characterization of the barley 5H chromatin using GISH and FISH

**Fig 1:**
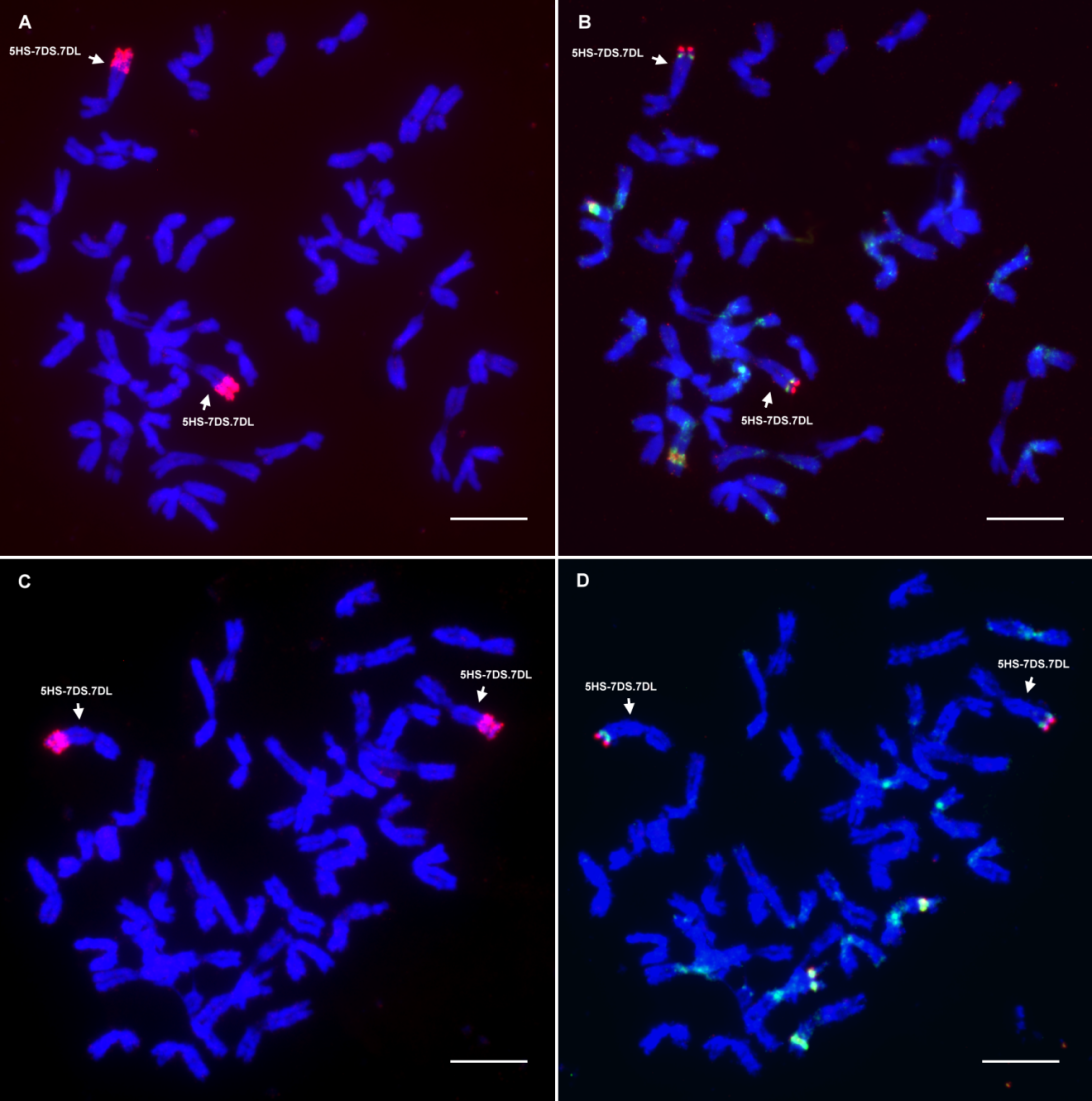
GISH and FISH analysis of the introgressed 5H barley chromatin in two genetic backgrounds. **(A)** GISH was performed on mitotic metaphase cells of the 5HS-7DS.7DL/Mv9kr1/Mv Bodri and (**C)** 5HS-7Ds.7DL/Mv9kr1 translocation lines. The 5HS barley chromatin was visualized with rhodamine (red). (**B)** FISH analysis was carried out on metaphase chromosomes in the 5HS-7DS.7DL/Mv9kr1/Mv Bodri and (**D)** 5HS-7DS.7DL/Mv9kr1 translocation lines. In the FISH images, the 5HS barley segment was analyzed using the HvT01 (red) and pTa71 (green) probes. The wheat chromosomes were counterstained with DAPI (blue). Arrows indicate the 5HS-7DS.7DL introgressed chromosome. Scale bar = 10 μm.

**Fig 2:**
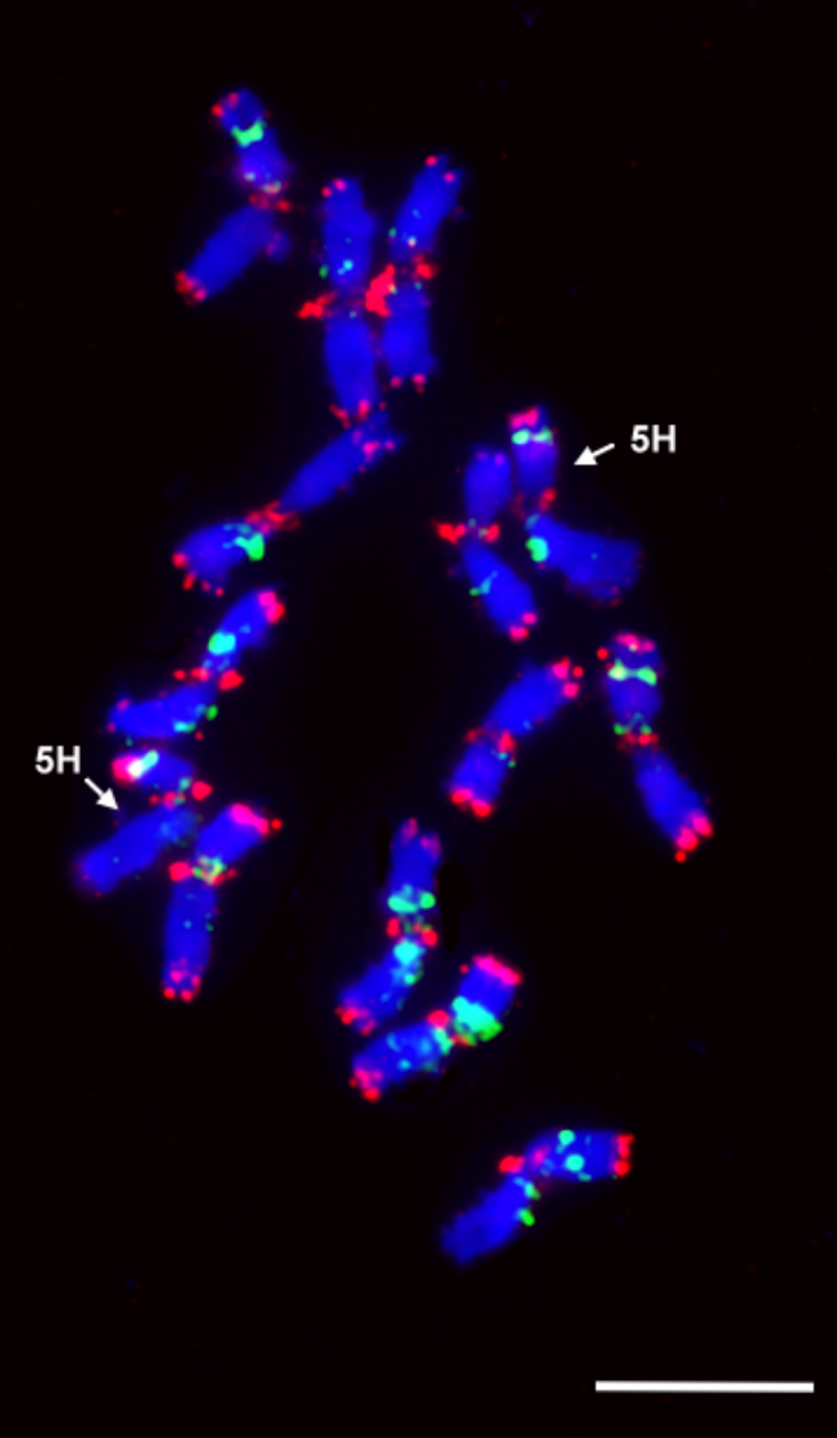
FISH pattern of barley (Igri) chromosomes. The 5H chromosomes could be identified and measured using repetitive DNA probes: HvT01 (red) in the subtelomeric region, pTa71 (green) in the satellite region, (GAA)_n_ in the pericentric region (green) and (AGGGAG)_n_ in the centromere (red). The 5H chromosomes are highlighted by arrows. Scale bar = 10 μm.

To study the structure of the introgressed 5HS chromatin and to obtain information about the missing part of the 5HS chromosome arm, the introgression lines were investigated with sequential FISH and GISH (Fig 1A–1D). The barley cultivar ‘Igri’ was also investigated with four repetitive DNA probes to obtain a reference karyotype for the 5H chromosome of barley (Fig 2). The hybridization signals of these probes served as cytogenetic landmarks for 5HS and their relative positions on the chromosome made it possible to calculate which part of the 5HS arm was missing in the translocation lines.

**Fig 3:**
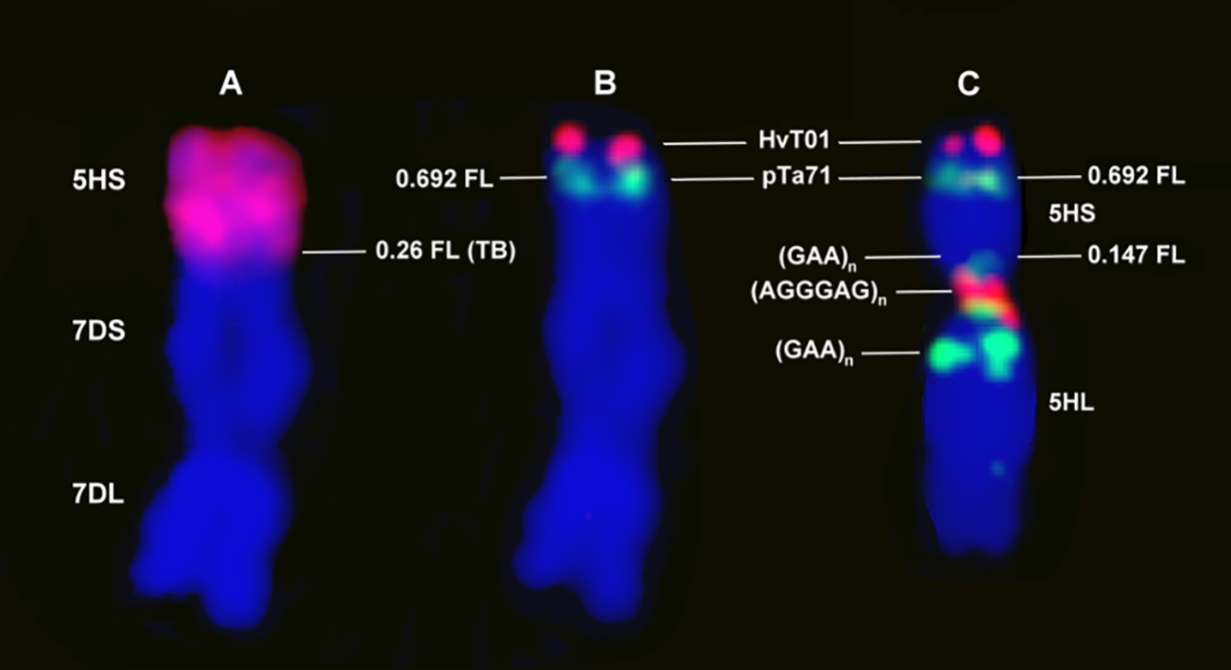
Representative picture for the determination of cytogenetic landmarks in the barley 5H chromatin. **(A)** The hybridization signals of GISH and (**B)** FISH on the introgressed barley chromatin were visualized in the 5HS-7DS.7DL/Mv9Kr1/Mv Bodri genotype. (**C)** The hybridization signals of FISH on the intact 5H chromosome indicate the physical position of the HvT01, pTa71, (GAA)_n_ and (AGGGAG)_n_ repetitive probes in the barley genotype Igri. The positions of the fluorescence signals were expressed as fraction length (FL) on the barley chromatin from the centromere (0 FL) to the telomere (1 FL). The length of the 5HS chromosome arm corresponds to the distance between the telomere and the centromeric repeat (AGGGAG)_n_. **(B, C)** The relative position of the satellite (pTa71 signal) is 0.692 FL, which is characterisitic of both intact and introgressed 5HS. **(C)** The region between the centromere-specific and pericentric (GAA)_n_ signals, which is missing from the 5HS-7DS.7DL translocation chromosome, represents the C-0.147 FL interval. **(A)** The relative position of the translocation breakpoint (TB) is 0.26 FL. The 1–0.26 FL region represents the transferred barley segment, accounting for 74% of the whole chromosome arm.

Measurements on 30 intact barley 5HS chromosomes showed that the region between the telomere and the satellite (pTA71 signal) represented 30.8% (FL: 0.692) of the entire 5HS arm and that between the centromere-specific and pericentric (GAA)_n_ signals 14.7% (FL: 0.147) (Fig 3C). Sequential GISH and FISH indicated that the pericentric (GAA)_n_ and centromeric (AGGGAG)_n_ signals were missing in the 5HS-7DS.7DL translocation (Fig 3A and 3B), while the distance between the telomere and the satellite (pTa71 signal), which accounted for 30.8% of the intact 5HS arm, was the same for the translocation lines as for the introgressed 5HS chromatin. It was possible to measure the total length of the introgressed barley chromatin visualized by GISH. As the relative position of the pTa71 signal on 5HS was the same in both the intact chromosome and the translocated segment, the relative position of the translocation breakpoints could be calculated from the total length of intact 5HS and the translocation segments. Based on measurements on 20 and 40 chromosomes respectively, the position of the translocation breakpoint of the introgressed 5HS segment relative to the whole 5HS chromosome arm was 0.27 FL ± 0.027 in 5HS-7DS.7DL/Mv9kr1 and 0.26 FL ± 0.025 in 5HS-7DS.7DL/Mv9kr1/Mv Bodri, indicating that the size of the introgressed barley segment (~74% of the whole 5HS arm) remained constant despite changes in the wheat genetic background, approximately 26% of the proximal part of the 5H short arm being absent from the translocated chromosome. The lack of the (GAA)_n_ and centromere-specific signals, also indicating that at least 14.7% of the proximal part of 5HS is missing, further supports these results.

### Molecular marker analysis

A total of about a thousand barley ESTs were analyzed using BLASTn (https://urgi.versailles.inra.fr/blast/blast.php). The query sequences (barley ESTs) were compared with the genomic sequences of wheat chromosomes. Ninety-five barley ESTs containing insertion/deletion regions (>7–8 bp) and showing at least 80% homology relative to wheat were chosen. Pairwise alignment was performed between the 95 selected ESTs and the corresponding wheat genomic sequences. Primer pairs were designed for the identified InDel regions in order to obtain markers amplifying the barley-specific fragments.

Primer sequences were developed for 35 of the 95 ESTs and these were validated by PCR on the control lines. The markers designed in the present study are listed in Table 1 together with their primer sequences and genetic positions. Six of the 35 EST-derived primer pairs either failed to produce a PCR amplicon in the barley genotypes or generated products non-polymorphic between the wheat and barley genotypes (Table 1).

**Table 1:** Validation of the EST-derived markers on the negative (Chinese Spring) and positive control lines (Betzes, Chinese Spring-Betzes 5H addition).

Marker^a^	Forward primer	Reverse primer	Barley EST name^b^	Position of the ESTs (cM)^b^	Barley-specific signal in the positive controls^c^
*Hv19657*	AGGACAACATCTGCTTCTCG	ATACAATGCACAAATCGCAA	19657	0	**+**
*Hv25687*	ACCTAGTGGTGGCGTACCT	AGGTCGGCGTGGTCATGC	25687	2.81	-
*Hv16076*	TAAGCCAAGGAGATCACGTC	TACTTTACAACAACGACGGC	16076	25.23	+
*Hv4027*	ACTGGAGGAGAAAGTGAAGC	GTGCGAGTGCGAGTGCTCGT	4027	26.28	-
*Hv1666*	GATCTCAAGTCCATTTCACG	TCGCAATGTGTCGCATTGCT	1666	29.90	-
*Hv16374*	CCGGTTTGTGTAGAACCAAG	CCAAATCTCAGCTCGCGTGA	16374	29.97	+
*Hv32679*	GCGGTTACTCATCACCACTC	CAGTTGCTCCAGCACAACTG	32679	29.97	-
*Hv48084*	GAGGAAGCTCCTTACCAAGC	CTACAACAAGCCTGATCGAT	48084	29.97	+
*Hv8574*	TGGAACTGATGTGAGCAACA	ATCTTACCAGAACCTCCCTT	8574	29.97	+
*Hv11893*	GTGGAGGACGAAGAGATGG	TCAACGCACAGCAGCAACAC	11893	29.97	+
*Hv1413*	AAGAAGAAGAAGCAGCAGCA	AGCAGCAGCAGCAACGGTGA	1413	29.97	+
*Hv4079*	TTAACTTTGCTCCAGGGATG	GCTTCCTATAGTTTGATTGG	4079	33.09	+
*Hv24684*	CAGCTGAGCTCTGATGATGA	GACAGCCAGATAAGATCCAC	24684	33.09	+
*Hv3528*	TAAGGCGTTTGACATGGAAT	TGGTCTGAACTGCTAGATTA	3528	39.97	+
*Hv19014*	ACTGCACATGGTTCTCGACT	AGCATGTTCGTAAGTGGTCG	19014	39.97	+
*Hv3781*	TCCCATAACTGGTTCAGCTC	GAGAGCCACACTGCATATAT	3781	41.64	+
*Hv34877*	TGACAATACAGGCTGGGACT	GTAGCAGTGTGCAGTTTGTA	34877	41.64	+
*Hv23691*	TAAGCTATGGCGCTGCTG	TTCTACTCATTCAGAGATTACAC	23691	50.27	+
*Hv7317*	GGCCGTATGAGTTGCTAAGA	ACTGGTGAGTGGTAAATATC	7317	50.27	+
*Hv4262*	TGCAGCAGTTCGCTCTACTA	AACTGTCCAGGTGGCAGCTG	4262	50.27	+
*Hv2530*	ACCTGTCATGGGTTTCCATA	TATGGTACTTCTGAGAAGTA	2530	50.72	-
*Hv14691*	GCTCTACAGGAAGCTCGTGA	CTCTTCTCTTTGCATCTTGA	14691	51.30	+
*Hv19799*	CTGTTCCACATAGGCAGCTT	CTCGGAGGTTTCGCCGGAAG	19799	51.30	+
*Hv2734*	GATGGTAGCTTCACCCGTTA	ATTCGCTAGCAAAGGACTAT	2734	51.30	+
*Hv8326*	CATCTTCCCATCAATGATCC	ACTCTATTGCTACGACAACG	8326	51.30	+
*Hv8011*	GCAACATTAACCAGGGTCAG	CTGTGTAGTATGAGAAATTC	8011	51.30	+
*Hv23495*	AAGCAGAAGAGAAGGCTGGT	GCGGGAGCTGGAGCTTGAGC	23495	51.30	+
*Hv7502*	GCAAGTGAAAGTGACCAAGA	TTCTGAACCTGAGCCGCG	7502	51.30	+
*Hv3949*	AGGAAGTCACATGCTCGTCT	GCATAAGAACTACCAGGTATT	3949	51.30	+
*Hv18070*	TATCCACGACATACCCAAGC	ACCTTAGCTTATGTGCTGGA	18070	51.30	+
*Hv18916*	GCTTTGGCTGATGTCATCTT	AGGTGCACCTGACCACTGCA	18916	51.30	+
*Hv26278*	TACGGCATGAAGAGGAAGAG	ATCTCCTCGGTCGATCTACA	26278	52.02	+
*Hv26490*	CCATTGTCGTTGTCATGGA	CCGGCGTTCGTGTGCCGCG	26490	52.60	+
*Hv18171*	TCGAGAGACTGAGACGGAGA	GGAGTGCCAGCAGTGAAACG	18171	57.98	+
*Hv15683*	GGATCATGTCGGTAGAGGAA	TTGATACGATACATAGAAGA	15683	59.74	-

^**a**^ The names of the markers correspond to the identification number of the source barley EST, the ‘Hv’ prefix refers to the abbreviation of the species (*Hordeum vulgare* L).

^**b**^ Name and position of the barley ESTs on the Genom Zipper of chromosome 5H [26].

^**c**^ +/-: presence or absence of the barley-specific PCR product on the positive control genotypes (Betzes, Chinese Spring-Betzes 5H addition).

**Fig 4:**
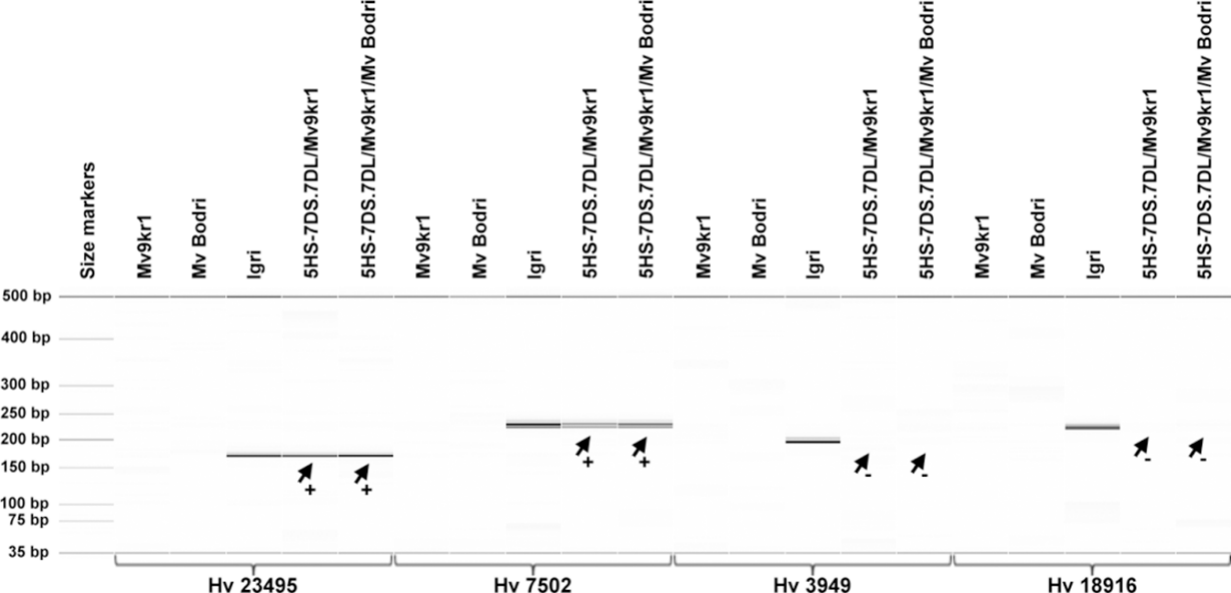
Digital capillary electrophoretic pattern of the barley EST-derived markers. The *Hv23495*, *Hv7502*, *Hv3949* and *Hv18916* markers specific for barley chromosome 5H were tested on wheat cultivars Mv9kr1 and Mv Bodri, barley cultivar Igri and the introgression lines 5HS-7DS.7DL/Mv9kr1 and 5HS-7DS.7DL/Mv9kr1/Mv Bodri. The plus or minus sign next to the arrow indicates the presence or absence of a barley-specific PCR product in the tested translocation lines. A 35–500 bp DNA ladder was used as a molecular-weight size marker to estimate the fragment size.

**Fig 5:**
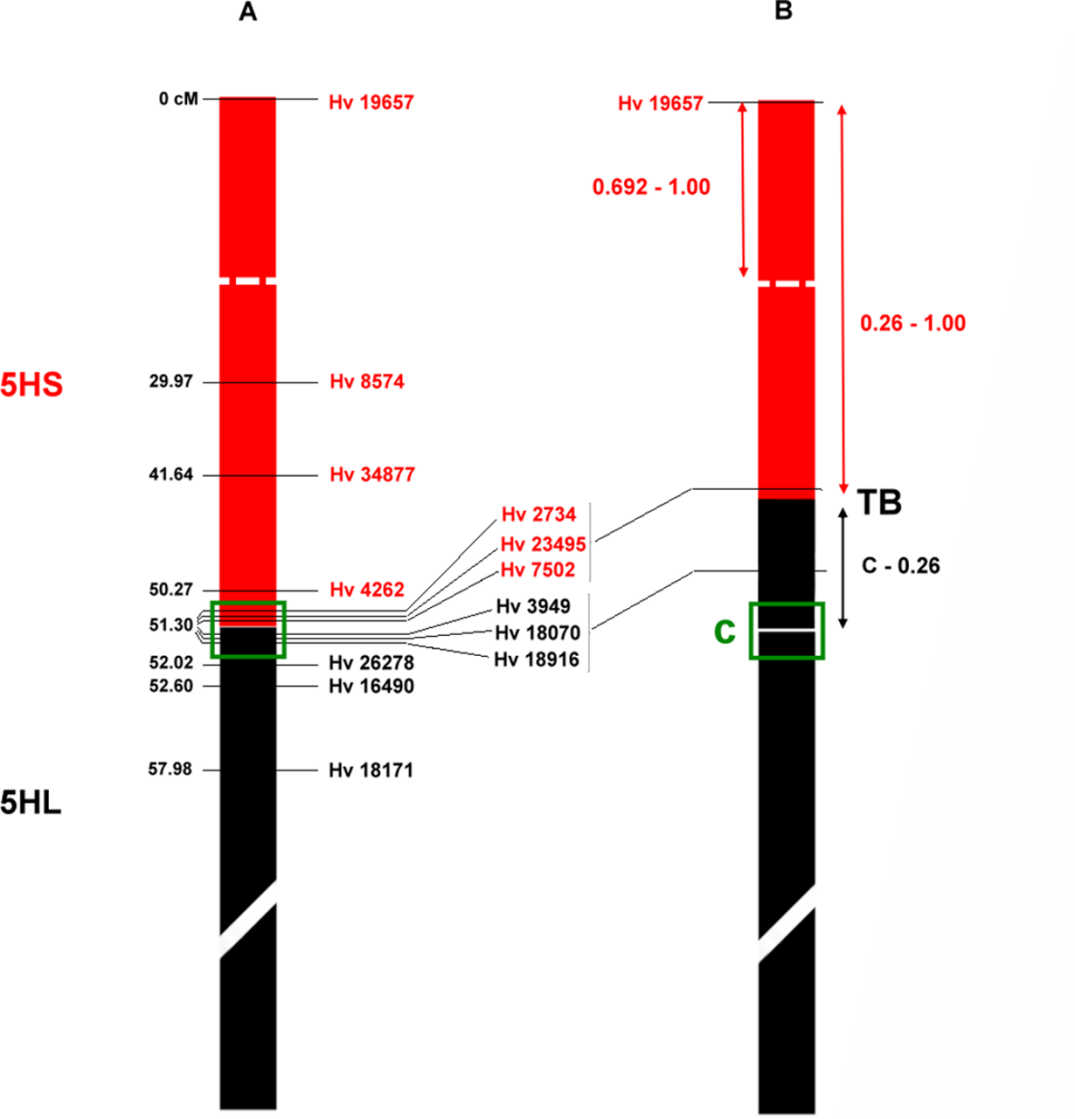
Comparison of genetic linkage and physical maps for the barley 5H chromosome. **(A)** The distribution of the EST-derived markers over the intact 5H chromosome of the barley genotype Igri is illustrated based on the high-resolution EST map found in Genom Zippers. The marker distance was expressed in centimorgans (cM) from the telomere of 5HS (0 cM) to the telomere of 5HL. (**B)** In the physical map of barley chromosome 5H the introgressed part of the 5HS chromosome arm and the molecular markers mapped on it are highlighted in red. The missing parts of 5HS and 5HL and the molecular markers specific to these regions are shown in black. The size of the introgressed and missing parts of 5H were expressed as fraction length (FL). The putative centromere position estimated by the cytogenetic landmarks is labeled C.

The remaining 29 markers were tested in the translocation lines and twenty-three were found to map within the 0–51.30 cM interval in both 5HS-7DS.7DL/Mv9kr1 and 5HS-7DS.7DL/Mv9kr1/Mv Bodri (Table 2). Three markers (*Hv3949*, *Hv18070*, *Hv18916*) located in the most proximal EST bin (51.30 cM) at the centromere were missing from the translocation lines and three (*Hv26278*, *Hv26490*, *Hv18171*) mapped to the proximal region of 5HL gave no PCR product in either of the translocation lines (Fig 4). These results indicate that the translocation breakpoint is located in the 51.3 cM region. The breakpoint was delimited between markers *Hv7502* and *Hv3949*, located at loci 653 and 725, respectively, which means that this part of the 51.3 cM region is equivalent to 73–74% of the 5HS chromosome arm (Fig 5). Marker analysis also revealed that the barley chromatin segment did not change in the modified wheat genetic background (Table 2).

**Table 2:** Analysis of the 5HS-7DS.7DL/Mv9kr1 and 5HS-7DS.7DL/Mv9kr1/Mv Bodri translocations with the negative (Mv9kr1, Mv Bodri) and positive control lines (Igri) using the molecular markers designed in the present study.

Marker	Barley EST loci^a^	Location on the 5H chromosome^a^	Barley-specific signal in the 5HS-7DS.7DL/Mv9kr1 translocation line^b^	Barley-specific signal in the 5HS-7DS.7DL/Mv9kr1/Mv Bodri translocation line^b^
*Hv19657*	4	**5HS**	**+**	**+**
*Hv16076*	81	**5HS**	+	+
*Hv16374*	118	**5HS**	+	+
*Hv48084*	123	**5HS**	+	+
*Hv8574*	124	**5HS**	+	+
*Hv11893*	126	**5HS**	+	+
*Hv1413*	133	**5HS**	+	+
*Hv4079*	137	**5HS**	+	+
*Hv24684*	138	**5HS**	+	+
*Hv3528*	165	**5HS**	+	+
*Hv19014*	166	**5HS**	+	+
*Hv3781*	176	**5HS**	+	+
*Hv34877*	180	**5HS**	+	+
*Hv23691*	466	**5HS**	+	+
*Hv7317*	473	**5HS**	+	+
*Hv4262*	489	**5HS**	+	+
*Hv14691*	542	**5HS**	+	+
*Hv19799*	547	**5HS**	+	+
*Hv2734*	621	**5HS**	+	+
*Hv8326*	646	**5HS**	+	+
*Hv8011*	650	**5HS**	+	+
*Hv23495*	653	**5HS**	+	+
*Hv7502*	653	**5HS**	+	+
*Hv3949*	725	**5HS**	-	-
*Hv18070*	770	**5HS**	-	-
*Hv18916*	945	**5HS**	-	-
*Hv26278*	979	**5HL**	-	-
*Hv26490*	987	**5HL**	-	-
*Hv18171*	1053	**5HL**	-	-

^**a**^ Barley EST loci and their distribution over the 5H barley chromosome according to Genom Zipper [26]

^**b**^ Comparison of the two introgressions using 5H-specific EST markers. +/-: presence or absence of the barley-specific signal in the 5HS-7DS.7DL translocation lines.

### Agronomic investigation

In order to clarify whether the translocation or the modification of the wheat genetic background had a greater influence on agronomic traits, the agronomic performance of the two translocation genotypes 5HS-7DS.7DL/Mv9kr1 and 5HS-7DS.7DL/Mv9kr1/Mv Bodri was compared, together with that of the parental wheat genotypes Mv9kr1 and Mv Bodri, in two field trials representing high-input (Table 3) and low-input (Table 4) conditions.

**Table 3:** Morphological traits of the two translocation lines and the parental wheat cultivars in the high-input field (Breeders Nursery) in the 2016–2017 growing season.

Genotype	Plant height (cm)	Length of the main spike (cm)	Spikes/plant	Seeds/mainspike	Spikelets/ main spike	Seeds/ spikelet	Seeds/plant	1000-Kernel weight (g)
Mv9kr1	94.1 ± 2.8^**a**^	10.9 ± 0.7^**a**^	10.8 ± 1.5^**b**^	58.1 ± 7.6^**a**^	23.0 ± 1.1^**a**^	2.5 ± 0.3^**b**^	512 ± 89^**b**^	38.7 ± 1.3^**a**^
Mv Bodri	83.2 ± 4.4^**b**^	10.4 ± 0.3^**a**^	10.9 ± 1.4^**b**^	65.4 ± 3.3^**a**^	21.6 ± 1.1^**ab**^	3.1 ± 0.2^**a**^	540 ± 68^**b**^	37.5 ± 2.8^**ab**^
5HS-7DS.7DL/Mv9kr1	78.1 ± 4.5^**c**^	8.6 ± 0.4^**b**^	13.1 ± 1.8^**a**^	47.4 ± 8.2^**b**^	18.1 ± 1.1^**c**^	2.6 ± 0.4^**b**^	550 ± 109^**b**^	35.3 ± 1.8^**b**^
5HS-7DS.7DL/Mv9kr1/ Mv Bodri	76.1 ± 3.3^**c**^	8.5 ± 0.6^**b**^	15.1 ± 2.2^**a**^	59.1 ± 7.6^**a**^	20.6 ± 1.6^**b**^	2.9 ± 0.3^**ab**^	681 ± 104^**a**^	38.4 ± 3.4^**a**^

Data represent mean ± standard deviation of 10 plants per genotype for each agronomic parameter. Different letters indicate significant differences between the genotypes at P<0.05, using Tukey’s post hoc test.

**Table 4:** Morphological traits of the two translocation lines and the parental wheat cultivars in the low-input field (Tükrös Nursery) in the 2015–2016 growing season.

Genotype	Plant height (cm)	Length of the main spike (cm)	Spikes/plant	Seeds/main spike	Spikelets/ main spike	Seeds/ spikelet	Seeds/plant	1000-Kernel weight (g)
Mv9kr1	88.8 ± 5.3^**a**^	9.0 ± 0.9^**a**^	5.1 ± 1.3^**b**^	37.6 ± 6.0^**ab**^	17 ± 1.3^**a**^	2.2 ± 0.4^**ab**^	146 ± 53^**b**^	39.5 ± 2.5^**a**^
Mv Bodri	61.8 ± 4.6^**c**^	8.5 ± 0.6^**ab**^	5.3 ± 1.5^**b**^	32.0 ± 11.4^**b**^	16.6 ± 1.5^**ab**^	2.0 ± 0.8^**b**^	158 ± 31^**b**^	39.1 ± 3.3^**ab**^
5HS-7DS.7DL/Mv9kr1	68.0 ± 3.5^**b**^	7.7 ± 0.4^**bc**^	6.7± 0.7^**ab**^	33.5 ± 4.6^**ab**^	14.2 ± 1.2^**c**^	2.4 ± 0.3^**ab**^	166 ± 16^**ab**^	36.4 ± 1.5^**b**^
5HS-7DS.7DL/Mv9kr1/ Mv Bodri	70.0 ± 3.5^**b**^	7.3 ± 0.6^**c**^	8.7 ± 2.6^**a**^	40.7 ± 3.6^**a**^	15.1 ± 1.1^**bc**^	2.7 ± 0.3^**a**^	211 ± 56^**a**^	38.7 ± 1.8^**ab**^

Data represent mean ± standard deviation of 10 plants per genotype for each agronomic parameter. Different letters indicate significant differences between the genotypes at P<0.05, using Tukey’s post hoc test.

In the two field experiments Tukey’s post-hoc analysis classified the plant height of both translocation lines in the same category, which differed from that of the wheat cultivars. Mv9kr1 was the tallest in both field trials. Mv Bodri was the shortest genotype in the low-input organic nursery, but grew much taller in the high-input area, where Mv Bodri was also taller than the two translocation genotypes. The length of the main spike was smaller in the introgression lines than in the wheat cultivars. 5HS-7DS.7DL/Mv9kr1/Mv Bodri had the highest number of spikes per plant at both locations, showing a significant difference compared with the parental lines. Both translocation lines had fewer spikelets per main spike than the wheat cultivars, indicating the effect of the introgressed barley chromatin or the loss of the 7DS wheat chromatin. It is important that the line 5HS-7DS.7DL/Mv9kr1/Mv Bodri had a higher number of seeds per main spike and spikelets per main spike than the initial introgression line indicating the positive effect of the Mv Bodri background under high imput conditions. The 5HS-7DS.7DL/Mv9kr1/Mv Bodri genotype had the most seeds per plant in both field trials, differing significantly from the other three lines. The 5HS-7DS.7DL/Mv9kr1/Mv Bodri genotype had higher thousand-kernel weight (TKW) than the 5HS-7DS.7DL/Mv9kr1 genotype in both field experiments. The two locations had similar average TKW values despite the different nutrient supplies.

**Fig 6:**
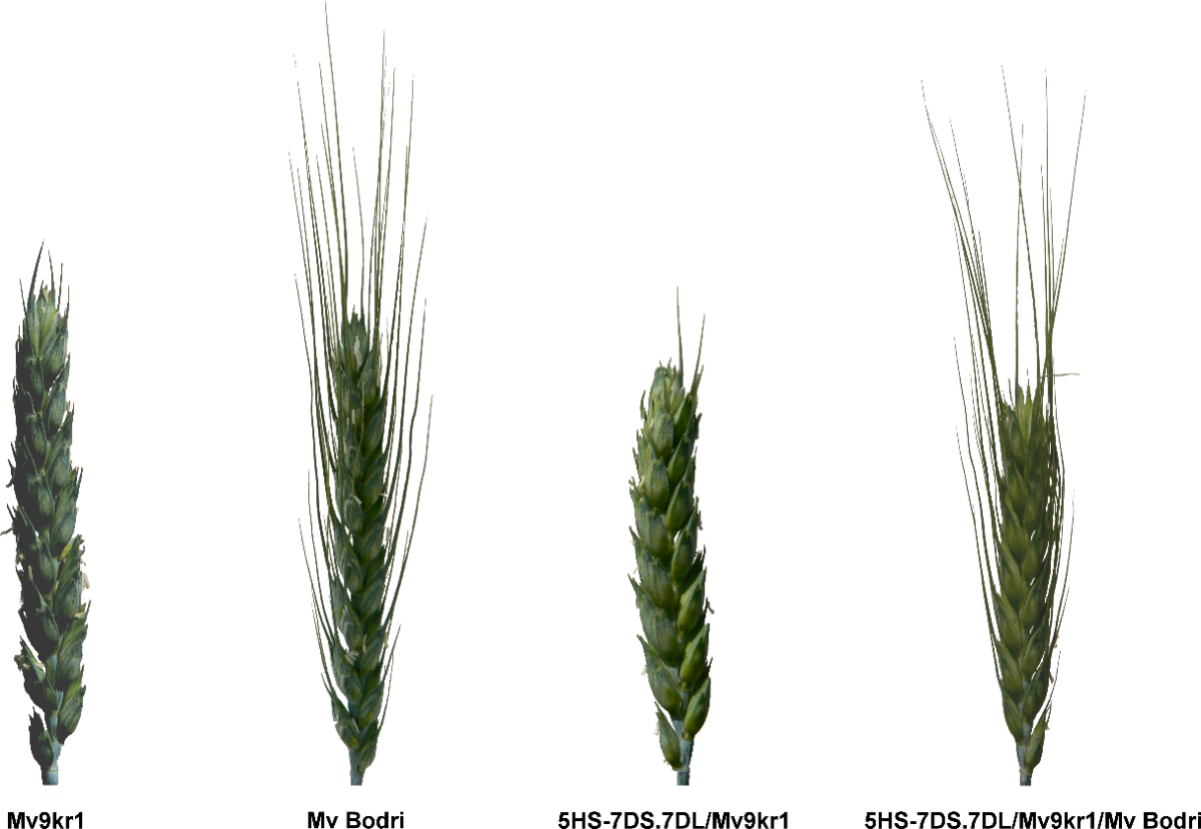
Spike morphology of the wheat cultivars and the wheat-barley translocation lines. The plants were grown in the Organic Nursery (Tükrös) in Martonvásár, Hungary in the 2015–2016 growing season.

The spike of 5HS-7DS.7DL/Mv9kr1/Mv Bodri had a dense structure with awned spikes, like those of the Mv Bodri wheat genotype. However, 5HS-7DS.7DL/Mv9kr1 had a main spike with apical awn stubs, like that of Mv9kr1 wheat (Fig 6).

## Discussion

As the 5H chromosome is eliminated most frequently in wheat x barley crossing programs [13,19,20], the importance of the 5HS-7DS.7DL translocation is underlined by the fact that this translocation has exhibited stable inheritance through 15 generations. In the present study, the 5HS chromosome segment of Igri barley was transferred into an elite wheat genetic background to obtain a genotype suitable for direct use in wheat breeding programs.

The structure of the introgressed barley chromatin was compared in the two different wheat genetic backgrounds, Mv9kr1 and Mv Bodri. Both molecular marker and molecular cytogenetic analysis confirmed that the size of the 5HS chromatin segment did not change when the wheat genetic background was modified. GISH and FISH determined the physical position of the translocation breakpoint at 0.26 FL of the whole 5HS arm. As the estimated length of the 5H short arm is 301 Mbp, compared with 5100 Mbp for the barley genome [39,40], it is estimated that the 74% of the whole 5HS arm transferred into wheat corresponds to 223 Mbp. The PCR analysis showed that 23 markers developed in the present study were mapped in the introgressed 1–0.26 FL (223 Mbp) bin, while three markers (*Hv3949*, *Hv18070*, *Hv18916*) were located in the missing C-0.26 FL region (78 Mbp) in both translocation genotypes. Interestingly, the translocation breakpoint was flanked by markers *Hv7502* and *Hv3949*, and seven of the ten markers, whose source ESTs were previously mapped within the centromeric region of 5HS (51.3–52.02 cM) [26,41], were located in the 1–0.26 FL region. These results indicate that the chromosomal breakage must be more distal from the centromere than was suggested by the genetic position of the source ESTs on the high-resolution EST map. In an earlier study Nagy et al. [22] delimited the position of this translocation breakpoint between the microsatellite markers *Bmag387* and *Bmag337*, so the present results also indicate that the *Bmag387* marker was located distally and the *Bmag337* marker proximally from the 0.26 FL position. It was also hypothesized earlier that the terminal region of 5HS had already been deleted in the barley genotype Igri due to a chromosomal rearrangement with a breakpoint near the satellite [22]. However, the telomere specific EST-derived marker (*Hv19657*) amplified a 5HS-specific fragment in the introgression lines and in Igri barley, indicating that the terminal segment of 5HS is present in the translocation.

This study supports the previous opinion that the linearly ordered virtual gene map of barley (Genome Zipper) is extremely useful in both fundamental and applied barley research [42]. The new EST-derived markers developed in this study will be used in wheat prebreeding programs aimed at shortening the introgressed 5HS segment. This marker development approach can also be applied for the marker saturation of 5HS chromosome regions containing desirable agronomic traits for wheat improvement. In this context, several QTLs related to abiotic stress tolerance were mapped on the 5HS barley chromatin. On the distal half of 5HS, two QTLs related to the osmotic adjustment of plant cells under water-limited and well-watered conditions were located [17]. Some physiological traits connected to the salt tolerance in barley were also mapped near the centromere of 5HS [18], such as a QTL affecting proline content and another related to the water-soluble carbohydrate content, which may facilitate heat or salt tolerance in the grain-filling period. Further studies will be needed in the future to prove that the introgressed 5HS segment has a real effect on the abiotic stress tolerance of wheat.

The present study also investigated how the presence of barley 5HS chromatin affects agronomic parameters as compared with the changes caused by the modification of the wheat genetic background. The number of spikes per plant is one of the most important parameters determining the yield of cereals. Both translocation lines had a higher number of spikes per plant than the wheat cultivars in both field trials, indicating that this parameter was influenced predominantly by the introgressed barley chromatin. The results for the effect of 5HS on tillering ability are in agreement with previous data published by Naz et al. [43], who identified five QTLs associated with tiller number per plant on barley chromosomes 1H, 2H, 4H and 5H, among which the strongest locus (*QTil*.*S42IL*.*5H*) was localized on 5H. Other studies also reported QTLs affecting tillering ability on chromosome 5H [44], such as the locus *spp-5H-1*, mapped close to the 50 cM region [45].

As no significant difference was observed in other yield components (seeds/main spike, seeds/spikelet and thousand-kernel weight) between 5HS-7DS.7DL/Mv9kr1/MvBodri and Mv Bodri, it can be concluded that increased tillering ability is the main yield component responsible for the higher yield (expressed as seeds/plant) in the 5HS-7DS.7DL/Mv9kr1/MvBodri genotype relative to the corresponding wheat genotype Mv Bodri under high-input field conditions. On the other hand, the increased fertility indicated by higher values of seeds per main spike and spikelets per main spike might have contributed to the increased yield of 5HS-7DS.7DL/Mv9kr1/MvBodri in comparison with 5HS-7DS.7DL/Mv9kr1, especially under high-input conditions, indicating the positive effect of the advanced wheat (Mv Bodri) genetic background. Further field trials using a larger plot area/genotype will be needed for a more exact comparison of yield parameters in 5HS-7DS.7DL/Mv9kr1/MvBodri and Mv Bodri.

Wheat genotype Mv Bodri was found to have lower plant height than Mv9kr1, which could be related to the fact that Mv Bodri carries the mutant allele of the semi-dwarfing gene *RhtD1b* on the short arm of 4D [46–48]. However, the deletion of the terminal region of 7DS in the translocation genotypes may also result in shorter plants. Khlestkina et al. [49] mapped the Ent-kaurenoic acid oxidase-coding (*KAO*) genes determining the gibberellin biosynthesis pathway and identified the *KAO-D1* gene on the terminal segment of 7DS. Later, Kruppa et al. [23] reported that the two microsatellite markers (*Xgwm1258*, *Xgwm1250*) flanking the *KAO-D1* locus were missing in the 5HS-7DS.7DL/Mv9kr1 genotype. Thus, the absence of the terminal region of the 7DS chromosome arm and consequently the absence of the *KAO-D1* gene may also contribute to the reduced plant height of 5HS-7DS.7DL/Mv9kr1/MvBodri.

## Conclusions

The improved tillering ability, together with the similar or better yield components under high- or low-input cultivation environments, respectively, indicate that the 5HS-7DS.7DL translocation in the Mv Bodri genetic background has small or negligible disadvantageous effects relative to the parental wheat Mv Bodri, which makes it a promising genotype for use in crossing and selection programs aimed at wheat improvement.

The exact size of the translocation was determined using *in situ* hybridization and molecular markers. As the new 5HS-7DS.7DL/Mv9kr1/MvBodri translocation, representing 74% of the whole 5HS arm, has stable inheritance in the wheat genetic background, this work represents an important step forward in the integration of the extremely instable barley chromosome 5H, carrying potential alleles for abiotic stress tolerance, into wheat breeding programs. The new EST-derived markers will be useful in pre-breeding programs to select wheat genotypes with introgressed 5HS chromatin or to identify genotypes with a shortened 5HS segment.

Altogether, the agronomic performance of the translocation line was improved by crossing it with a high-yielding wheat cultivar and the 5HS segment had a positive effect on tillering ability, which will be further tested in field experiments in the future. The new translocation line promise to accelerate functional genomic studies on barley chromosome 5H and the new molecular markers will support pre-breeding and breeding research on wheat, which will be required to meet the future challenges of food security and sustainable agriculture.

## References

[1] Farkas A, Molnár I, Kiss T, Karsai I, Molnár-Láng M. Effect of added barley chromosomes on the flowering time of new wheat/winter barley addition lines in various environments. Euphytica. 2014;195: 45–55. doi: 10.1007/s10681-013-0970-7

[2] Toojinda T, Baird E, Booth A, Broers L, Hayes P, Powell W, et al Introgression of quantitative trait loci (QTLs) determining stripe rust resistance in barley: An example of marker-assisted line development. Theor Appl Genet. 1998;96: 123–131. doi: 10.1007/s001220050718

[3] Vales MI, Schön CC, Capettini F, Chen XM, Corey AE, Mather DE, et al Effect of population size on the estimation of QTL: A test using resistance to barley stripe rust. Theor Appl Genet. 2005;111: 1260–1270. doi: 10.1007/s00122-005-0043-y 16179997 10.1007/s00122-005-0043-y

[4] Darkó É, Barnabás B, Molnár-Láng M. Characterization of newly developed wheat/barley introgression lines in respect of aluminium tolerance. Am J Plant Sci. 2012;3: 1462–1469. doi: 10.4236/ajps.2012.310176

[5] Darkó É, Janda T, Majláth I, Szopkó D, Dulai S, Molnár I, et al Salt stress response of wheat–barley addition lines carrying chromosomes from the winter barley “Manas.” Euphytica. 2015;203: 491–504. doi: 10.1007/s10681-014-1245-7

[6] Darkó É, Gierczik K, Hudák O, Forgó P, Pál M, Türkösi E, et al Differing metabolic responses to salt stress in wheat-barley addition lines containing different 7H chromosomal fragments. PLoS One. 2017;12: 1–20. doi: 10.1371/journal.pone.0174170 28328973 10.1371/journal.pone.0174170 PMC5362201

[7] Szopkó D, Darkó É, Molnár I, Kruppa K, Háló B, Vojtkó A, et al Photosynthetic responses of a wheat (Asakaze)–barley (Manas) 7H addition line to salt stress. Photosynthetica. 2017;55: 317–328. doi: 10.1007/s11099-016-0241-7

[8] Karsai I, Mészáros K, Láng L, Bedő Z. Identification of chromosome regions involved in the genetic regulation of tillering in barley (*Hordeum vulgare* L.). Acta Agron Hungarica. 2006;54: 15–23. doi: 10.1556/AAgr.54.2006.1.2

[9] Türkösi E, Farkas A, Aranyi NR, Hoffmann B, Tóth V, Molnár-Láng M. Improvement of the agronomic traits of a wheat–barley centric fusion by introgressing the 3HS.3BL translocation into a modern wheat cultivar. Genome. NRC Research Press; 2014;57: 601–607. doi: 10.1139/gen-2014-0187 25806585 10.1139/gen-2014-0187

[10] Jukanti AK, Fischer AM. A high-grain protein content locus on barley (Hordeum vulgare) chromosome 6 is associated with increased flag leaf proteolysis and nitrogen remobilization. Physiol Plant. 2008;132: 426–439. doi: 10.1111/j.1399-3054.2007.01044.x 18333996 10.1111/j.1399-3054.2007.01044.x

[11] Cseh A, Soos V, Rakszegi M, Türkösi E, Balázs E, Molnár-Láng M. Expression of HvCslF9 and HvCslF6 barley genes in the genetic background of wheat and their influence on the wheat β-glucan content. Ann Appl Biol. 2013;163: 142–150. doi: 10.1111/aab.12043

[12] Molnár-Láng M, Linc G. Wheat-barley hybrids and introgression lines. In: Molnár-Láng M, Ceoloni C, Doležel J, editors. Alien Introgression in Wheat: Cytogenetics, Molecular Biology, and Genomics. Springer International Publishing; 2015 p. 315–345. doi: 10.1007/978-3-319-23494-6

[13] Islam AKMR, Shepherd KW, Sparrow DHB. Isolation and characterization of euplasmic wheat-barley chromosome addition lines. Heredity (Edinb). 1981;46: 161–174. doi: 10.1038/hdy.1981.24

[14] Szakács É, Molnár-Láng M. Development and molecular cytogenetic identification of new winter wheat–winter barley (“Martonvásári 9 kr1”–“Igri”) disomic addition lines. Genome. 2007;50: 43–50. doi: 10.1139/g06-134 17546070 10.1139/g06-134

[15] Molnár-Láng M, Novotny C, Linc G, Nagy ED. Changes in the meiotic pairing behaviour of a winter wheat-winter barley hybrid maintained for a long term in tissue culture, and tracing the barley chromatin in the progeny using GISH and SSR markers. Plant Breed. Blackwell Publishing Ltd; 2005;124: 247–252. doi: 10.1111/j.1439-0523.2005.01097.x

[16] Szakács É, Molnár-Láng M. Identification of new winter wheat–winter barley addition lines (6HS and 7H) using fluorescence in situ hybridization and the stability of the whole “Martonvásári 9 kr1”–“Igri” addition set. Genome. 2010;53: 35–44. doi: 10.1139/g09-085 20130747 10.1139/g09-085

[17] Teulat B, Borries C, This D. New QTLs identified for plant water status, water-soluble carbohydrate and osmotic adjustment in a barley population grown in a growth-chamber under two water regimes. Theor Appl Genet. 2001;103: 161–170. doi:https://doi.org/10.1007/s001220000503

[18] Siahsar BA, Narouei M. Mapping QTLs of physiological traits associated with salt tolerance in Steptoe × Morex doubled haploid lines of barley at seedling stage. J Food, Agric Environ. 2014;8: 2.

[19] Koba T, Handa T, Shimada T. Efficient production of wheat-barley hybrids and preferential elimination of barley chromosomes. Theor Appl Genet. 1991;81: 285–292. doi: 10.1007/BF00228665 24221254 10.1007/BF00228665

[20] Molnár-Láng M, Kőszegi B, Linc G, Galiba G, Sutka J. Chromosome instability of wheat/barley ditelosomic addition lines in tissue culture. Cereal Res Commun. 1996;24: 275–281. doi: 10.2307/23784213

[21] Molnár-Láng M, Linc G, Logojan A, Sutka J. Production and meiotic pairing behaviour of new hybrids of winter wheat (Triticum aestivum) × winter barley (Hordeum vulgare) Genome. NRC Research Press Ottawa, Canada; 2000;43: 1045–1054. doi: 10.1139/g00-079 11195337

[22] Nagy E. D, Molnár-Láng M, Linc G, Láng L. Identification of wheat-barley translocations by sequential GISH and two-colour FISH in combination with the use of genetically mapped barley SSR markers. Genome. 2002;45: 1238–1247. doi: 10.1139/g02-068 12502270 10.1139/g02-068

[23] Kruppa K, Sepsi A, Szakács É, Röder MS, Molnár-Láng M. Characterization of a 5HS-7DS.7DL wheat-barley translocation line and physical mapping of the 7D chromosome using SSR markers. J Appl Genet. Springer Berlin Heidelberg; 2013;54: 251–258. doi: 10.1007/s13353-013-0152-2 23749613 10.1007/s13353-013-0152-2

[24] Molnár-Láng M, Linc G, Sutka J. Transfer of the recessive crossability allele kr1 from Chinese Spring into the winter wheat variety Martonvásári 9. Euphytica. 1996;90: 301–305. doi: 10.1007/BF00027480

[25] Mayer KFX, Taudien S, Martis M, Simkova H, Suchankova P, Gundlach H, et al Gene content and virtual gene order of barley chromosome 1H. Plant Physiol. 2009;151: 496–505. doi: 10.1104/pp.109.142612 19692534 10.1104/pp.109.142612 PMC2754631

[26] Mayer KFX, Martis M, Hedley PE, Simková H, Liu H, Morris JA, et al Unlocking the barley genome by chromosomal and comparative genomics. Plant Cell. 2011;23: 1249–1263. doi: 10.1105/tpc.110.082537 21467582 10.1105/tpc.110.082537 PMC3101540

[27] Mikó P, Löschenberger F, Hiltbrunner J, Aebi R, Megyeri M, Kovács G, et al Comparison of bread wheat varieties with different breeding origin under organic and low input management. Euphytica. 2014;199: 69–80. doi: 10.1007/s10681-014-1171-8

[28] Jiang J, Friebe B, Gill BS. Recent advances in alien gene transfer in wheat. Euphytica. Kluwer Academic Publishers; 1994;73: 199–212. doi: 10.1007/BF00036700

[29] Schubert I, Shi F, Fuchs J, Endo TR. An efficient screening for terminal deletions and translocations of barley chromosomes added to common wheat. Plant J. Blackwell Science Ltd; 1998;14: 489–495. doi: 10.1046/j.1365-313X.1998.00125.x

[30] Gerlach WL, Bedbrook JR. Cloning and characterization of ribosomal RNA genes from wheat and barley. Nucleic Acids Res. Oxford University Press; 1979;7: 1869–1885. doi: 10.1093/nar/7.7.1869 537913 10.1093/nar/7.7.1869 PMC342353

[31] Pedersen C, Linde-Laursen IB. Chromosomal locations of four minor rDNA loci and a marker microsatellite sequence in barley. Chromosom Res. Kluwer Academic Publishers; 1994;2: 65–71. doi: 10.1007/BF01539456 10.1007/BF01539456 8162323

[32] Vrána J, Kubaláková M, Šimková H, Číhalíková J, Lysák MA, Doležel J. Flow sorting of mitotic chromosomes in common wheat (Triticum aestivum L.). Genetics. 2000;156: 2033–2041. 11102393 10.1093/genetics/156.4.2033 PMC1461381

[33] Hudakova S, Michalek W, Presting GG, Hoopen RT, Santos KD, Jasencakova Z, et al Sequence organization of barley centromeres. Nucleic Acids Res. Oxford University Press; 2001;29: 5029–5035. doi: 10.1093/nar/29.24.5029 11812833 10.1093/nar/29.24.5029 PMC97617

[34] Reader SM, Abbo S, Purdie KA, King IP, Miller TE. Direct labelling of plant chromosomes by rapid in situ hybridization. Trends Genet. 1994;10: 265–6. Available: http://www.ncbi.nlm.nih.gov/pubmed/7940753 7940753 10.1016/0168-9525(90)90007-s

[35] Molnár-Láng M, Linc G, Friebe BR, Sutka J. Detection of wheat-barley translocations by genomic in situ hybridization in derivatives of hybrids multiplied in vitro. Euphytica. Kluwer Academic Publishers; 2000;112: 117–123. doi: 10.1023/A:1003840200744

[36] Linc G, Friebe BR, Kynast RG, Molnar-Lang M, Kőszegi B, Sutka J, et al Molecular cytogenetic analysis of Aegilops cylindrica host. Genome. 1999;42: 497–503. doi: 10.1139/g98-151 10382296

[37] Cseh A, Kruppa K, Molnár I, Rakszegi M, Doležel J, Molnár-Láng M, et al Characterization of a new 4BS.7HL wheat–barley translocation line using GISH, FISH, and SSR markers and its effect on the β-glucan content of wheat. Genome. 2011;54: 795–804. doi: 10.1139/g11-044 21919737 10.1139/g11-044

[38] Spannagl M, Martis MM, Pfeifer M, Nussbaumer T, Mayer KF. Analysing complex Triticeae genomes—concepts and strategies. Plant Methods. BioMed Central; 2013;9: 35 doi: 10.1186/1746-4811-9-35 24011260 10.1186/1746-4811-9-35 PMC3847682

[39] Doležel J, Greilhuber J, Lucretti S, Meister A, Lysák MA, Nardi L, et al Plant genome size estimation by flow cytometry: inter-laboratory comparison. Ann Bot. Oxford University Press; 1998;82: 17–26. doi: 10.1093/oxfordjournals.aob.a010312

[40] Suchánková P, Kubaláková M, Kovářová P, Bartoš J, Číhalíková J, Molnár-Láng M, et al Dissection of the nuclear genome of barley by chromosome flow sorting. Theor Appl Genet. 2006;113: 651–659. doi: 10.1007/s00122-006-0329-8 16810504 10.1007/s00122-006-0329-8

[41] Close TJ, Bhat PR, Lonardi S, Wu Y, Rostoks N, Ramsay L, et al Development and implementation of high-throughput SNP genotyping in barley. BMC Genomics. 2009;10: 582 doi: 10.1186/1471-2164-10-582 19961604 10.1186/1471-2164-10-582 PMC2797026

[42] Mascher M, Stein N. Genetic anchoring of whole-genome shotgun assemblies. Front Genet. 2014;5: 208 doi: 10.3389/fgene.2014.00208 25071835 10.3389/fgene.2014.00208 PMC4083584

[43] Naz AA, Arifuzzaman M, Muzammil S, Pillen K, Léon J. Wild barley introgression lines revealed novel QTL alleles for root and related shoot traits in the cultivated barley (Hordeum vulgare L.). BMC Genet. 2014;15: 107 doi: 10.1186/s12863-014-0107-6 25286820 10.1186/s12863-014-0107-6 PMC4200126

[44] Chutimanitsakun Y, Nipper RW, Cuesta-Marcos A, Cistué L, Corey A, Filichkina T, et al Construction and application for QTL analysis of a Restriction Site Associated DNA (RAD) linkage map in barley. BMC Genomics. 2011;12: 4 doi: 10.1186/1471-2164-12-4 21205322 10.1186/1471-2164-12-4 PMC3023751

[45] Peighambari SA, Samadi BY, Nabipour A, Charmet G, Sarrafi A. QTL analysis for agronomic traits in a barley doubled haploids population grown in Iran. Plant Sci. 2005;169: 1008–1013. doi: 10.1016/j.plantsci.2005.05.018

[46] Börner A, Plaschke J, Korzun V, Worland AJ. The relationships between the dwarfing genes of wheat and rye. Euphytica. Kluwer Academic Publishers; 1996;89: 69–75. doi: 10.1007/BF00015721

[47] McCartney CA, Somers DJ, Humphreys DG, Lukow O, Ames N, Noll J, et al Mapping quantitative trait loci controlling agronomic traits in the spring wheat cross RL4452 × “AC Domain.” Genome. NRC Research Press Ottawa, Canada; 2005;48: 870–883. doi: 10.1139/g05-055 16391693 10.1139/g05-055

[48] Gulyás G, Bognár Z, Láng L, Rakszegi M, Bedõ Z. Distribution of dwarfing genes (Rht-B1b and Rht-D1b) in Martonvásár wheat breeding materials. Acta Agron Hungarica. 2011;59: 249–254. doi: 10.1556/AAgr.59.2011.3.8

[49] Khlestkina EK, Kumar U, Röder MS. Ent-kaurenoic acid oxidase genes in wheat. Mol Breed. 2010;25: 251–258. doi: 10.1007/s11032-009-9326-3

